# Microbiology of Ethiopian Traditionally Fermented Beverages and Condiments

**DOI:** 10.1155/2020/1478536

**Published:** 2020-02-14

**Authors:** Bikila Wedajo Lemi

**Affiliations:** Department of Biology, College of Natural Sciences, Arba Minch University, Arba Minch, P.O. Box 21, Ethiopia

## Abstract

Globally, fermented beverage and condiments are made by using different conventional practices, raw materials, and microorganisms. This paper presents the available literature review on the technology and microbiology of traditional Ethiopian beverages and condiment products. Traditional fermented beverage and condiment products have essential vitamins, minerals, enzymes, and antioxidants that are all enhanced through the process of traditional fermentation practices. In Ethiopia, fermented beverage and condiment products have practiced in a long history. During the production of traditional fermented beverage and condiment products, controlled natural fermentation process with the absence of starter cultures are used to initiate it. Moreover, the preparation of many traditionally fermented beverage and condiment products is still practiced in a household art, thereby a wide variety of fermented beverages and condiments are consumed in Ethiopia. In conclusion, the review discusses the nature of the beverage and condiment preparation, poor traditional household processing, and the extent and limitation of scientific work done so far and suggests some recommendations to limit the problem in Ethiopia.

## 1. Background

Ethiopia is a country rich in cultural diversity. The variety of foods and beverages, processed and consumed among the various ethnic groups are indicators of this diversity. Ethiopia is one of the countries where a wide variety of traditional fermented foods and beverages are prepared and consumed. Fermented products prepared from plant and animal sources play an important role in human food due to changes in the physical, chemical, and nutritional properties by microorganisms [1].

Fermented beverages constitute a major part of the diet of traditional African homes serving as from inebriating drinks to weaning foods; in addition, these beverages also play a major role in social functions in which they are consumed in different occasions such as marriage, naming, and rain making ceremonies [2], at festivals and social gatherings, and at burial ceremonies and settling disputes. They are also used as medicines for fever and other ailments by adding barks or stems of certain plants [3]. Traditional fermented beverages are those which are indigenous to a particular area and have been developed by people of that area themselves using age-old techniques from locally available (mostly home-grown) raw materials. The traditionally fermented products are manufactured using only rudimentary equipment such as earthen vessels, and handling and consumption often takes place under conditions of poor hygiene [4]. Fermented beverages produced from cereals are usually referred to as beers, while those produced from fruits, milk sap, honey, or molasses are classified as wines [5, 6].

Traditional fermented condiments are among the various traditional fermented foods, which are produced by microbial fermentations under highly variable conditions in different parts of the world [6]. Such food products are not consumed alone, but are added as a condiment to make the food more tasty and enjoyable [7]. Condiments constitute significant proportion of African diets where they serve as flavorsome and culinary components in various dishes [8]. In recent years, the use of fermented food condiments and flavoring agents are becoming popular in the diets of many nations. Apart from the fact that those condiments improve sensory properties of foods and vitamins [9], some of them contain antioxidants and medicinal properties that provide health benefits [10], detoxification of antinutrient factors [11], and contain proteins [12], fatty acids, and good sources of gross energy [8, 13].

Ethiopian local fermented foods and beverages are products of acid-alcohol type of fermentation. These include “*injera*,” “*ergo*,” “*ititu*,” “*ayib*,” “*qibe*,” “*arrera*,” “*kocho*,” “*tella*,” “*siljo*,” “*datta*,” “*awaze*,” “*borde*,” “*tej*,” “*areki*,” “*cheka*,” “*azo*,” “*keribo*,”and “*korefe*.” Of these “*tella*,” “*cheka*,” “*tej*,” “*borde*,” “*areki*,” “*keribo*,”and “*korefe*” are among the varieties of fermented beverages consumed in Ethiopia, while “*awaze*,” “*datta*,” “*siljo*,” and “*azo*” are among the varieties of traditionally fermented condiments known in Ethiopia. Therefore, the aim of this paper was to review microbiological studies made by various researchers on fermentation, other processing methods, and microbial safety of traditional Ethiopian beverages and condiments.

## 2. Traditionally Fermented Beverages

### 2.1. “*Tella*” Fermentation

**Figure 1:**
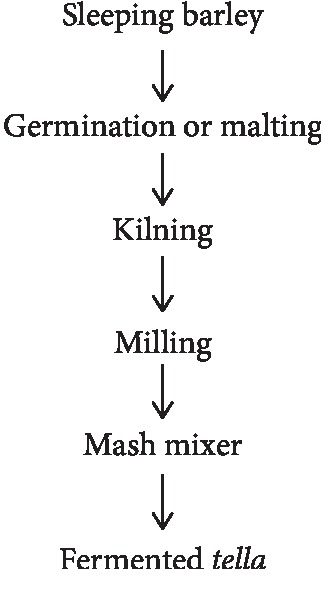
Flow chart of traditional preparation of *tella* [18].

“*Tella*” is a popular Ethiopian traditional beverage, which is made from diverse ingredients. “*Tella*” has various vernaculars in the various regions and is based on substrates such as barley, wheat, maize, millet, sorghum, “*teff*,” or other cereals [14–16]. The earthen clay pot container (“Insera” in Amharic or “Gaanii” in Afaan Oromo) is washed with water and leaves of “grawa” (*Vernonia amygdalina*) and water several times. Then, the cleaned container is inverted over smoking wood fragments of “weyra” (*Olea europaea*) for about 10–15 minutes. This will remove microorganisms sensitive to wood smoke and adds the desired flavor to product. “Bikil” (malt), the source of amylase from corn or barley or wheat grain is moistened in a container and left to germinate for about three days and finally sun-dried. The gesho plant (*Rhamnus prinoides*), local hops, which is different from hop (*Humulus lupulus*) is widely cultivated in Ethiopia and is available in the dried form in the local market. Kita is broken into small pieces. Barley flour is separately toasted on a metal pan sprinkling water on it during toasting until it turns dark brown. This is called enkuro. The color of “*tella*,” which may vary from light yellow to dark brown, is determined by the extent of baking kita or toasting enkuro. This mixture of enkuro, the rest of the germinated grains (bikil), some gesho, and water are added to the container. The mixture is kept covered overnight, after which more water is added, and the container is kept sealed for 5 to 7 days, until the beverage is ready. “*Tella*” can be kept for 10 to 12 days [14, 17]. The fermenting organisms composed of *Saccharomyces* spp., (mostly *S*. *cerevisiae*) and *Lactobacillus* spp., (mostly *Lactobacillus pastorianumi*) (Figure 1).

### 2.2. “*Areki*” Fermentation

“*Areki*” is a distilled, colorless, clear, traditional alcoholic beverage in which fermented products are prepared in almost the same way as “*tella*” except that the fermentation mass in this case is more concentrated [15, 19]. “*Areki*” fermentation product is known as Yereki-tinsis which is prepared by mixing powdered gesho leaves and powdered bikil (1 : 2 ratios) with water to give a mixture of free flowing consistency and will be put aside to ferment for about five days. Traditionally *areki* is classified as terra-areki and dagim-areki. The term dagim in Amharic refers to “second time” and designates that it is distilled second time, whereas the term terra in Amharic refers to “ordinary” [19, 20]. The alcohol content of terra-areki was reported to be 34.09% (v/v) [20] and varies between 22.0–28.0% (v/v) [21]. Dagim-areki is redistilled to give terra-areki that have higher alcohol content with the average of around 45% (v/v) [21]. It was also reported to have a mean value of 46.6% (v/v) ethanol content [20]. Since the government has no control over the production of locally brewed alcoholic drinks, it is difficult to estimate the amount of alcohol production and consumption in Ethiopia [21].

### 2.3. “*Cheka*” Fermentation

**Figure 2:**
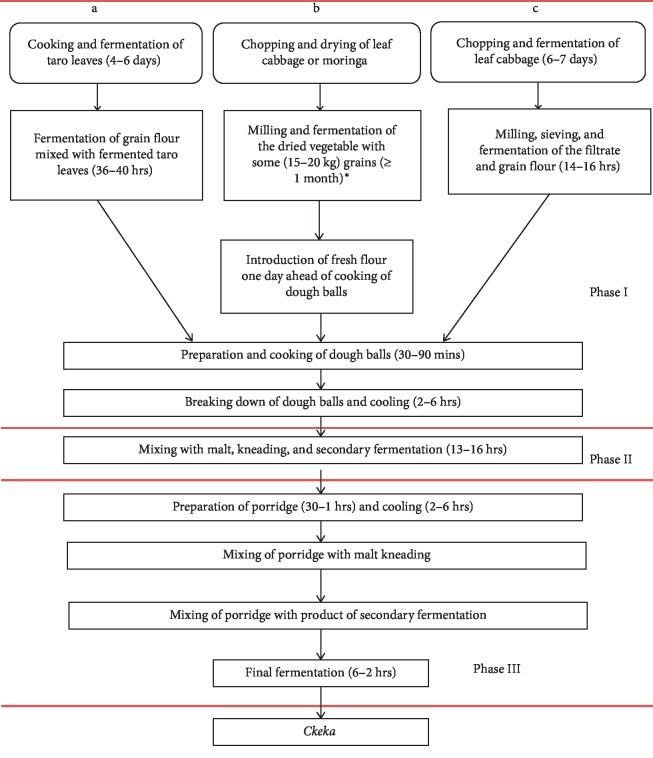
Flow chart for *cheka* preparation in Konso and Dirashe districts [22].

“*Cheka*” is a cereal and vegetable-based fermented beverage which is consumed in Southwestern parts of Ethiopia [22]. *Cheka* is mainly prepared from cereals such as sorghum (*Sorghum bicolor*), maize (*Zea mays*), and vegetables such as leaf cabbage (*Brassica* spp.), moringa (*Moringa stenoptella*), and decne (*Leptadenia hastata*). In some localities, few households also use dried edible leftovers of *injera*, *kitta*, or *kurkufa*. The processes of *cheka* preparation are very complex and vary among households, villages, and localities. Three types of *cheka* are produced in the study districts such as hiba (parshota), chaqa (fasha), and menna (poh-kedha or madhot). The duration of *cheka* fermentation varies from 12 hours for menna to months for parshota [22] (Figure 2).

### 2.4. “*Tej*” Fermentation

**Figure 3:**
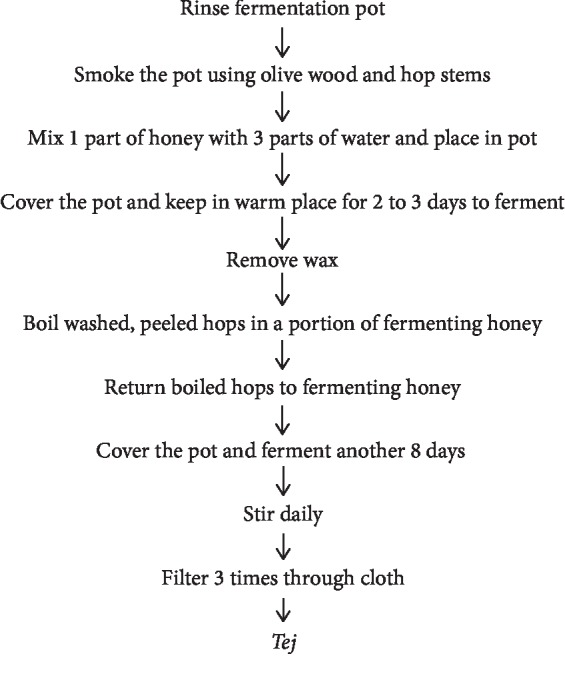
Flow chart of traditional preparation of *tej* [16].

“*Tej*” is a home-processed and commercially available honey wine. Some *tej* producers also include different concoctions such as barks, roots of some plants, and herbal ingredients to improve flavor or potency of *tej*. During the preparation of *tej*, the fermentation pot is seasoned by smoking over glowing of *Olea africana* and gesho (*Rhamnus prinoides*) and put back to fermenting [16]. Honey, which may contain various impurities including wax, is mixed with water and placed in the smoked pot. The pot is covered and fermented continuously for five more days, in warmer weather, or for 15–20 days, in cooler environments. Fermentation of *tej*, relies on the microorganisms (lactic acid bacteria and yeast) present in the substrates, fermentation vats, and equipment. Their metabolic products contribute to acidity and also add distinctive flavor and aroma to the fermenting material. Major yeast species in *tej* were *Saccharomyces cerevisiae*, *Kluyveromyces bulgaricus*, *Debaromyces phaffi*, and *Kluyveromyces veronae*. The lactic flora consisted of *Lactobacillus*, *Streptococcus*, *Leuconostoc*, and *Pediococcus* species [16, 23] (Figure 3).

### 2.5. “*Borde*” Fermentation

**Figure 4:**
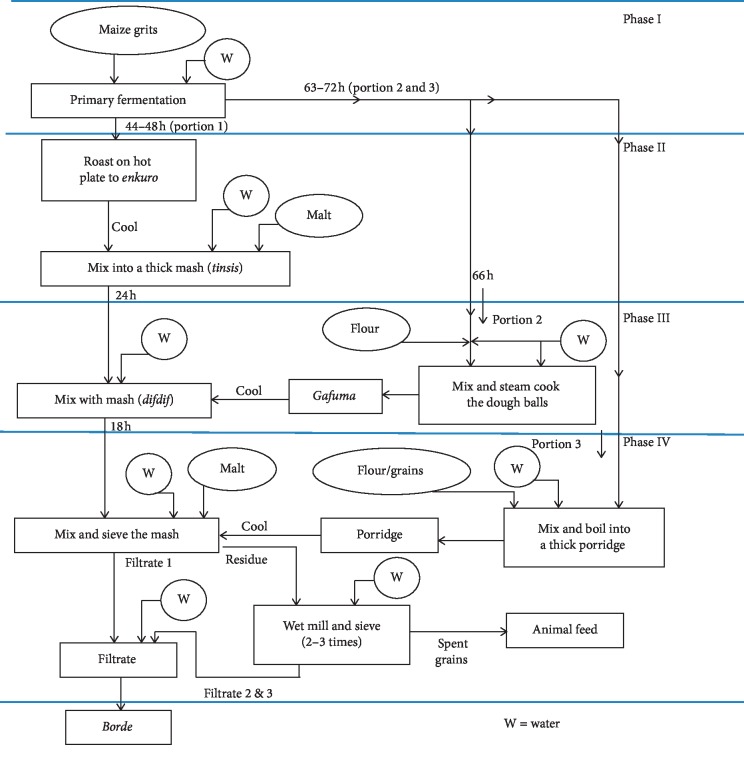
Flow charts of traditional preparation of *borde* [25].

“*Borde*” is a multipurpose cereal-based (maize (*Zea mays*), barley (*Hordeum vulgare*), wheat (*Triticum sativum*), finger millet (*Eleusine coracana*), sorghum (*Sorghum bicolor*), and *tef* (*Eragrostis tef*)) traditionally fermented beverage and is widely consumed in the southern and western parts of Ethiopia. It is produced by spontaneous fermentation using a simple equipment. *Borde* is a whitish-grey-to-brown-coloured beverage, with a bushy consistency and a sweet-sour taste [24, 25]. *Borde* is considered to be a low-alcoholic beverage even though the duration of its fermentation is long enough (4 days) to result in a considerable accumulation of ethanol [15] (Figure 4).

### 2.6. “*Shamita*” Fermentation

**Figure 5:**
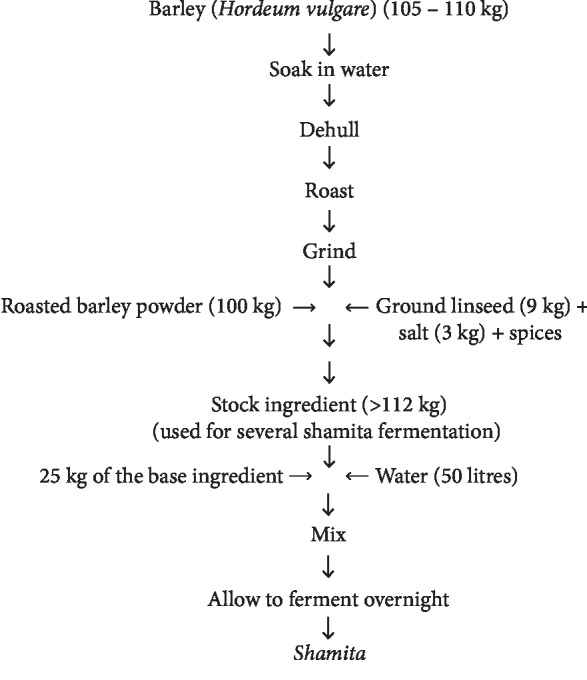
Flow chart of laboratory *shamita* fermentation process [26].

“*Shamita*” is another traditional beverage of Ethiopia, which is low in alcohol content, made by overnight fermentation of mainly roasted barley flour and consumed as meal replacement. *Shamita* is a widely consumed beverage in different regions of Ethiopia. It has a thick consistency, and most people who cannot afford a reasonable meal consume it as a meal replacement [26]. The microbes (LAB and yeast) liable for fermentation are mostly from back slopping using a small amount of *shamita* from previous fermentation as well as from the ingredients and equipment. These microorganisms make the product a good source of microbial protein. However, *shamita* has poor keeping quality because of these high numbers of live microorganisms and becomes too sour about four hours after being ready for consumption [27, 28] (Figure 5).

### 2.7. “*Keribo*” Fermentation

**Figure 6:**
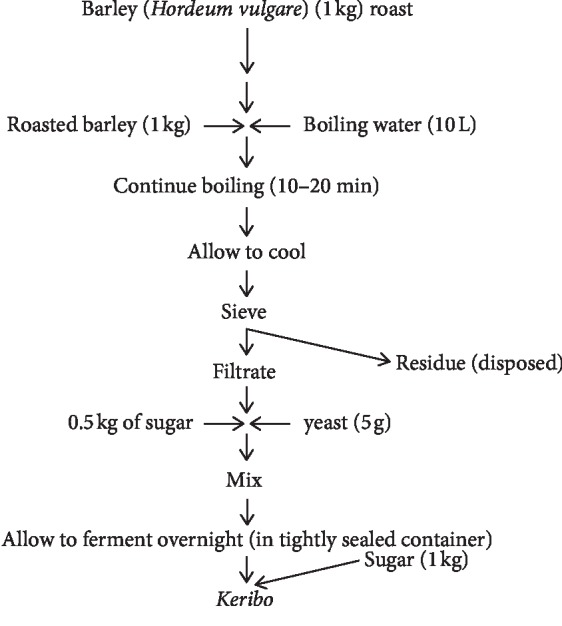
Flow chart of traditional *keribo* fermentation [29].

Among the various fermented beverages, *keribo* is a traditional fermented beverage produced mainly from barley and sugar in different parts of Ethiopia. The fermented *keribo* beverage is being served on holidays, wedding ceremony, and also serves as sources of income of many households. The popularity of this traditionally fermented beverage is more reflected among the religious groups and those do not like alcoholic drinks because as it is considered as a non- or low-alcoholic beverage [25]. Moreover, the safety consideration of Ethiopian foods and beverages has shown the possibility of isolating some foodborne pathogens from some fermented products. However, there is no scientifically documented information both on the microbiology and safety of *keribo* preparation. Therefore, *keribo* fermentation needs further investigation in the near future [29] (Figure 6).

### 2.8. “*Korefe*” Fermentation

**Figure 7:**
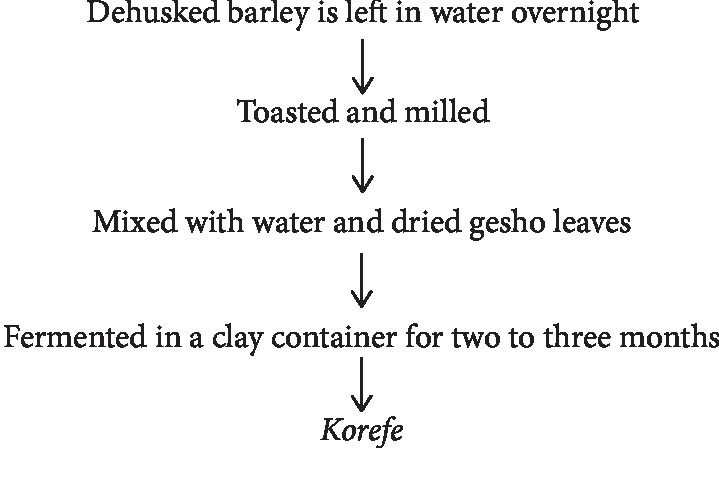
Flow chart of traditional *korefe* fermentation [31].

*Korefe* is the name of the traditional indigenous fermented beverage made in Begemder province among the Koumant ethnic group in Ethiopia. Dehusked barley is left in water overnight and after that toasted and milled. It is mixed with water and dried gesho leaves and fermented in a clay container for two to three months. When the beverage is needed, a small quantity of the mixture is taken, more water is added, and after a day's fermentation, the beverage is ready for consumption [30]. Yeasts are organisms which are responsible for the fermentation process *korefe*. The average alcoholic contents of *korefe* ranged from 4.08–5.44% v/v. The mean ethanol contents have significant variations among samples of the same types. It might be due to the differences in preparation and fermentation conditions such as temperature, aeration, and actions of the microorganisms [30, 31] (Figure 7).

## 3. Fermented Condiments

### 3.1. “*Awaze*” Fermentation

It is known that, fermented food, beverage, and condiment products are commonly produced throughout the world. Different countries of Africa's protein-rich food ingredients are often fermented to make condiments which result from the microbial fermentations of vegetable-spice mixtures [32]. *Awaze* is common in the north and central Ethiopia and is often used to flavor slice raw or roasted meat and other traditional pancakes. The major ingredient for *awaze* preparation is red sweet pepper (*Capsicum annum*). The spices added to it include garlic (*Allium sativum*), ginger (*Zingiber officinale*), sweet basil (*Ocimum sanctum*), rue (*Ruta chalepensis*), cinnamon (*Cinamommum zylanicum*), clove (*Eugenia caryophyla*), Ethiopian caraway (*Trachyspermum copticum*), Ethiopian cardamom (*Aframomum anguistifolium*), and salt. *Awaze* fermentation starts by whipping a portion of the ground pepper-spice ingredient with warm water until it attains a thick consistency and left to ferment at ambient temperatures. The most dominant microorganisms in fermentation of *awaze* are aerobic mesophilic microflora such as *Bacillus* spp. and Lactic acid bacteria (LAB) [33, 34].

### 3.2. “*Datta*” Fermentation

Datta (also called qotchqotcha) is a condiment of similar use as that of *awaze* mainly in the southern part of Ethiopia and is consumed with other items on the basis of their desirable aromas and flavors resulted from the microbial fermentations of vegetable-spice mixtures. The major substrate in making of *datta* is the small chili pepper (*Capsicum frutescence*) at its green stage. Garlic and ginger, small amounts of fresh sweet basil and seeds of rue, were peeled, washed, and cut into small pieces and mixed together [34, 35].

The mixed ingredients were manually wet-milled on a flat smooth traditional stone-mill into a greenish paste and transferred into a 500 ml screw-cap bottle to ferment at 20 to 25°C. Lactic acid bacteria (LAB) initiated fermentation, and later the homolactic LAB started and dominated the fermentation for the first 2 days and the heterolactic LAB took over thereafter [34].

Challenge studies on *datta* fermentation with *Salmonella typhimurium* [34] and *E*. *coli* O157: H7 [35] showed that the fermenting condiments had strong bactericidal properties against the test strains. The fermenting product, when stored at ambient temperature, also had a fast inhibitory property against *E*. *coli* O157: H7, although the pathogen survived for more than seven days at refrigeration storage [35].

### 3.3. “*Siljo*” Fermentation

**Figure 8:**
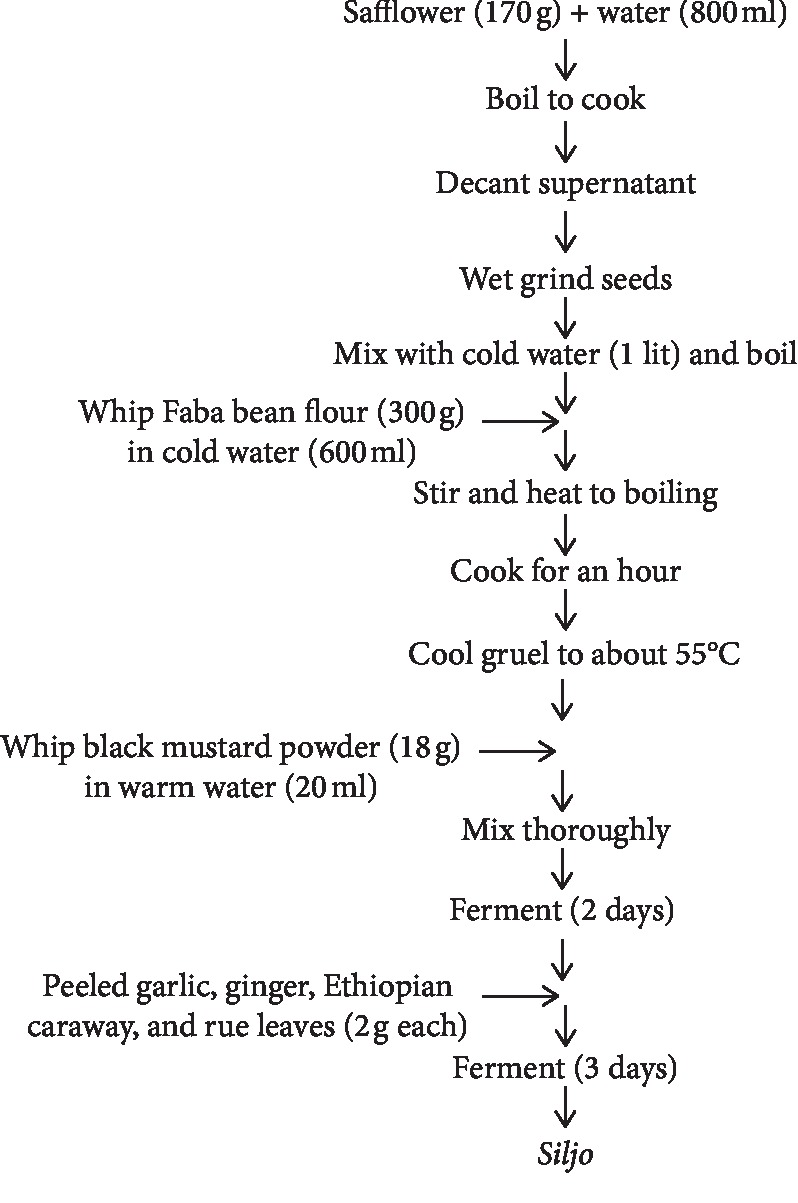
Flow chart of *siljo* fermentation [28].

A typical example of legume fermentation practiced in Ethiopia is *siljo* fermentation. *Siljo* is one of the traditionally fermented condiments of Ethiopia made up of safflower (*Carthamus tinctorius*) extract and faba bean (*Vicia faba*) flour. It is a popular condiment during the long fasting period before Easter [32]. The black mustard powder, added after cooking the mixture of the safflower and faba bean, helps as source of starter microorganisms. It contained *Lactobacillus acidophilus*, *L*. *plantarum*, and *L*. *delbrueckii* and the yeasts *Saccharomyces cerevisiae*, *Rhodotorula glutinis*, *Yarrowia lipolytica*, and *Saccharomyces rouxii*. The fermentation was, but, initiated and later dominated by *L*. *plantarum* and *L*. *acidophilus*. The fermented product has protein and fat contents of 28 and 25%, respectively [25, 28] (Figure 8).

### 3.4. “*Azo*” Fermentation

**Figure 9:**
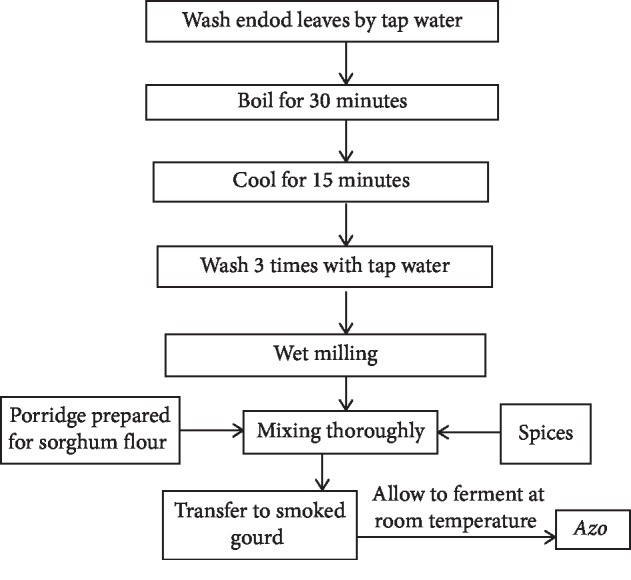
Schematic representation of steps involved for the preparation of *Azo* in laboratory [36].

*Azo* is a traditionally fermented semisolid condiment prepared from cereal flour and leaves of endod in northwest Ethiopia Tigray regional state. Of course, the way of processing and ingredients added for the preparation of this condiment differ from one community to other. Cereal flours and fresh leaves of endod are primary ingredients used for *Azo* preparation along with different variety spices, namely rue, garlic, ginger, korerima, black cumin, black mustards, and as minor ingredients for flavor enhancement. *Azo*-fermented product is consumed during Easter fasting season. Fermented condiments are used as taste enhancers in many traditional dishes [8]. Regarding *azo* fermentation is a few scientific work undertaken so far (Figure 9).

## 4. Safety Aspects of Traditionally Fermented Foods

Because many fermented foods are produced using microorganisms, the risk of toxin contamination is high. During natural fermentations, food-poisoning flora and coliforms may also grow with the fermented food product. These microorganisms need to be eliminated to make fermented foods safe for consumption [37]. Several factors contribute to the safety of fermented foods: (i) soaking and cooking—washing, soaking, and cooking treatments reduce the in situ microbial contaminants; (ii) salting—various fermented foods are made with the addition of salt, which acts as a preservative; (iii) acid formation—many indigenous fermentations are carried out by acid-producing microorganisms, where these organic acids act as preservatives or as bacteriostatic agents. An inhibitory pH for bacterial growth is considered to be 3.6 to 4.1; (iv) antibiotic production—molds used in some traditional fermentations produce antimicrobial glycopeptides; (v) low moisture content—in the case of SSF processes, the low water activity may be an important preservative factor. Despite these factors, it has been reported that the sanitary quality of some oriental fermented foods is poor [38, 39]. Safe products are usually obtained when the following recommendations are observed: (a) appropriate soaking of the beans in acid at a low pH; (b) adequate cooking time; (c) using hygienic conditions during production, handling, and storage; and (d) good refrigeration of products (5°C) between production and consumption.

## 5. Conclusion

In conclusion, Asia is well-known for its exotic traditionally fermented food and beverage products produced using a wide range of raw materials, microorganisms, and fermentation processes. The indigenous methods of fermentation were aimed to preserve and balance the availability of food sources. Furthermore, many scientific research studies have exhibited promising and sustainable opportunities related to these traditionally fermented food products. The nutritional values of fermented foods are related to a unique group of microflora that may enhance health benefits directly through the interaction with the host or indirectly through metabolites synthesized during fermentation. The bioactive compounds and other interactions within fermented food can add novel flavors to food and impart potential health benefits. However, future studies need to be conducted in order to explore various aspects of fermented food products, such as the determination of biomarkers for fermented food health benefits, safety concerns related to these products, and the bioaccessibility of microbial metabolites.
